# Visual Short-Term Memory for Coherent and Sequential Motion: A rTMS Investigation

**DOI:** 10.3390/brainsci11111471

**Published:** 2021-11-06

**Authors:** Andrea Pavan, Filippo Ghin, Gianluca Campana

**Affiliations:** 1 Department of Psychology, University of Bologna, Viale Berti Pichat 5, 40127 Bologna, Italy; 2 School of Psychology, University of Lincoln, Brayford Wharf East, Lincoln LN5 7AY, UK; Filippo.Ghin@uniklinikum-dresden.de; 3 Department of Child and Adolescent Psychiatry, Cognitive Neurophysiology, Faculty of Medicine of the TU Dresden, Fetscherstraße 74, 01307 Dresden, Germany; 4 Dipartimento di Psicologia Generale, University of Padova, Via Venezia 8, 35131 Padova, Italy; gianluca.campana@unipd.it; 5 Human Inspired Technology Research Centre, University of Padova, Via Luzzati 4, 35121 Padova, Italy

**Keywords:** visual short-term memory, repetitive transcranial magnetic stimulation, visual memory precision, serial memory effects

## Abstract

We investigated the role of the human medio-temporal complex (hMT+) in the memory encoding and storage of a sequence of four coherently moving random dot kinematograms (RDKs), by applying repetitive transcranial magnetic stimulation (rTMS) during an early or late phase of the retention interval. Moreover, in a second experiment, we also tested whether disrupting the functional integrity of hMT+ during the early phase impaired the precision of the encoded motion directions. Overall, results showed that both recognition accuracy and precision were worse in middle serial positions, suggesting the occurrence of primacy and recency effects. We found that rTMS delivered during the early (but not the late) phase of the retention interval was able to impair not only recognition of RDKs, but also the precision of the retained motion direction. However, such impairment occurred only for RDKs presented in middle positions along the presented sequence, where performance was already closer to chance level. Altogether these findings suggest an involvement of hMT+ in the memory encoding of visual motion direction. Given that both position sequence and rTMS modulated not only recognition but also the precision of the stored information, these findings are in support of a model of visual short-term memory with a variable resolution of each stored item, consistent with the assigned amount of memory resources, and that such item-specific memory resolution is supported by the functional integrity of area hMT+.

## 1. Introduction

Visual short-term memory (VSTM) is an active store of incoming visual information that is required for the completion of certain tasks and cognitive needs and allows to hold information for a few seconds [1]. The VSTM is closely connected to an individual’s cognitive ability, can be investigated at the level of the neural circuits, and is easily testable with specific procedures (see [2] for a review). VSTM involves the activity of many cortical areas such as frontal, occipital, posterior, and parietal cortices [3].

Behavioral research in humans showed that different attributes of visual stimuli are stored in visual short-term memory (VSTM). For example, McKeefry et al. [4] showed that stimulus characteristics such as orientation and direction of motion were stored in the VSTM. Pashler [5] and Vogel et al. [6] suggested that we can store up to four visual objects at one given time in the visual short-term memory, though Eng et al. [7] showed that the capacity of the VSTM was influenced by the perceptual complexity of the sample memory display. However, if there was a long time frame for stimulus encoding, then more would be remembered. It was concluded that, while complexity affects the capacity of the VSTM, it does not determine it.

Studies on human and non-human primates using a masking-delayed paradigm showed that VSTM for moving stimuli is maximally affected by the masking pattern, when it is presented during the retention interval 0.2 s after the offset of the memory sample, and it has the same physical characteristics as the memory pattern (i.e., same motion direction, spatial location, and speed [8,9,10]. Further evidence has been also provided by brain imaging and brain stimulation studies, demonstrating that implicit VSTM for simple stimulus attributes relies on the same (low-level) cortical areas that process such attributes [11,12,13]. In the motion domain, there is evidence that information about speed and direction can be accurately stored [14,15,16] and that the VSTM for such stimulus attributes is sensitive to early interference by an intervening masking stimulus. Pasternak and Zaksas [8] investigated the retention of motion in two macaque monkeys that were required to compare two sequentially presented coherent random dot kinematograms (RDKs) separated by a temporal delay [17,18,19]. A random-motion (noise) mask was introduced during the delay period. The mask interfered with performance only when it was presented in the same location as of the test, approximately 0.2 s after the start of the delay period, and when its speed matched that of the remembered sample. Therefore, the representation of coherent motion information in VSTM preserves direction, speed, and spatial position and is most vulnerable to visual interference shortly after the completion of the sensory encoding phase [8,9]. This selectivity of masking effects resembles the selectivity shown in humans for spatial frequency, temporal frequency, and speed of gratings [4,15,20], suggesting that VSTM for these stimulus attributes might share similar mechanisms and neural substrates. Using a similar paradigm to that of Pasternak and Zaksas [8], Pavan et al. [10] showed that the visual mask mainly interfered with participants’ performance when displayed 0.2 s after the offset of the sample and when its direction and speed matched that of the remembered sample. These results support the notion that the memory representation of global motion is selective for direction and speed, being compromised by intervening directional stimuli presented immediately after the encoding phase.

In this study, we used repetitive transcranial magnetic stimulation (rTMS) to investigate the role of the human complex MT (hMT+), an area involved in visual motion processing [21,22]. In two distinct experiments, we aimed to interfere with the encoding and retention of sequential coherent motion information by delivering repetitive TMS (rTMS) over the left hMT+. In the first experiment, participants had to memorize the direction of a motion sequence composed of four RDKs presented in rapid succession. The task was to report whether a probe RDK presented after the motion sequence and, after a 3 s retention interval, was contained in the to-be-remembered motion sequence. In similar change detection tasks, in which participants were asked to detect the presence of suprathreshold changes among an array of items (including color, shape, motion direction, etc.) after a short retention period, results showed that observers were accurate for array sizes of up to three to four items [1,6,23,24]. Based on these results, item-limit models of memory argue for a VSTM capacity of three to four independent memory slots, each storing information about an integrated visual object. On the other hand, Kawasaki et al. [25] found that this capacity seemed to be even lower for motion direction as the average capacity limit was limited to about two slots. In experiment 1, rTMS was delivered either early or late during the retention interval and after the offset of the rapid motion sequence. The goal was to test whether rTMS delivered over the retention interval interfered with the encoding (for early TMS) and/or the retention (for late rTMS) of sequential coherent motion information. As previous studies found that the capacity of VSTM is up to four integrated visual objects [1,26], we expected early and late rTMS to interfere with the encoding and retention. Additionally, we also assessed the presence of serial effects (i.e., primacy/recency), and how rTMS affects motion sensitivity depending on the spatial position of the target in the sequence.

In the second experiment, we assessed whether rTMS interferes with the precision of the memory trace for motion direction and serial position in the temporal sequence. To this purpose, we employed the same sequence of coherent RDKs and assessed the precision of the to-be-remembered direction of the motion stimulus depending on their serial position in the temporal sequence. This experiment is based on the more recent view that visual memory capacity is not fixed by the number of objects, as suggested by the limited capacity memory model, instead, it is a resource that is also limited but can be spread out and shared across all items available within the current visual scene [27,28,29,30]. This implies that the precision of remembering an item is dependent on how much of the resource it demands, though memory precision is expected to decrease as the number of visual items increases. This approach led to the dynamic resource model of visual short-term memory, which suggests that the resolution with which the visual object is stored in memory corresponds to the specific amount of memory resource assigned to that item [27,28,29,31,32,33]. Additionally, based on these dynamic models of VSTM, the performance also depends on memory for object locations or serial positions in a temporal sequence. In the second experiment, we expect that rTMS delivered during the retention interval mainly interferes with the precision of VSTM for motion direction when the target RDK is presented at intermediate serial positions in the motion sequence (i.e., either 2 or 3).

## 2. Experiment 1

### 2.1. Methods

#### 2.1.1. Participants

Two of the authors (AP and FG) and eleven naïve observers took part in this experiment. All participants had normal or corrected-to-normal visual acuity. The viewing was binocular. Each participant completed a questionnaire to assess for seizure, implanted metal objects, heart problems, or any other psychiatric or neurological disease. Written informed consent was obtained from each participant. Methods were carried out following the World Declaration of Helsinki [34]. Data were collected at the University of Lincoln (UK) and the present study was approved by the local Ethics Committee of the University of Lincoln (protocol number: PSY1718170).

#### 2.1.2. Apparatus

Stimuli were generated using MATLAB Psychtoolbox [35,36,37] and displayed on a 20-inch HP p1230 monitor with a refresh rate of 85 Hz, with a screen resolution of 1280 × 1024 pixels. Each pixel subtended 0.032 deg (i.e., ~ 1.9 arc min). The mean luminance was 37.5 cd/m^2^, with minimum and maximum luminance set to 0.08 cd/m^2^ and 74.6 cd/m^2^, respectively. A gamma-corrected lookup table was used so that luminance was a linear function of the digital representation of the image. Observers sat in a darkened room at 57 cm from the screen. The participant’s head was stabilized by using a chin-head rest.

#### 2.1.3. Stimuli

Stimuli were random dot kinematograms (RDKs) consisting of 200 white dots (dot diameter = 0.063 deg) presented within a circular aperture with a diameter of 9.4 deg (density = 2.85 dots/deg^2^). All the dots moved along translational trajectories with 100% coherence. The dots moved on a grey background (mean luminance 37.5 cd/m^2^) at a speed of approximately 5 deg/s [38]. The dots had a Weber contrast of 0.99. Dots had also a limited lifetime; after 0.047 s each dot vanished and was replaced by a new dot at a different randomly selected position within the circular window. Dots appeared and disappeared asynchronously on the display to avoid any flicker [39,40]. Limited lifetime and asynchronous dot displays were implemented to avoid attentional tracking of single moving elements. In addition, moving dots that traveled outside the circular window were replaced by a new dot at a different random location within the circular window, thus always maintaining the same dot density [38,41]. Dots could move towards one of eight directions (cardinal and intercardinal directions: 0°, 45°, 90°, 135°, 180°, 225°, 270°, 315°). For each motion sequence, we randomly chose four directions with the constraint that they were always different (i.e., the minimum pairwise angular separation between directions was 45°).

#### 2.1.4. Repetitive TMS

To localize the target cortical areas to stimulate and set the TMS intensity, the phosphene threshold was estimated individually for each participant. rTMS stimulation was delivered through a MagPro X100 stimulator (Medtronic, Denmark) with a figure-eight coil of 90 mm. Participants wore a swimming cap. The target stimulation site was localized in all observers by using predetermined coordinates: — 3 cm dorsal to inion and 5 cm leftward from there—for the localization of hMT+. Our decision to stimulate the left hMT+ was due to previous evidence which showed, using TMS, a quite strong lateralization of motion perception in the left hemisphere [42,43]. Moreover, this localization technique has been used in previous studies [11,12,43,44,45,46,47,48,49,50,51,52,53] and provides a localization that is consistent with fMRI localizers [50,54]. In fact, in our previous rTMS study [50] we showed that hMT+ localization based on the craniometric procedure mostly overlaps with that based on neuro-navigation. In general, all the studies reported showed that TMS applied over hMT+ can produce moving or flickering phosphenes. Thus, the induction of moving or flickering phosphenes is considered a reliable method that can prevent confusing hMT+ with other adjacent cortical areas.

An adaptive procedure (i.e., rapid estimation of phosphene thresholds [REPT] [55]) was used to estimate the rTMS intensity for which participants perceived phosphenes in 60% of the trials with eyes closed and blindfolded. The adaptive staircase consisted of 30 trials. Phosphene thresholds were estimated delivering a cycle of three pulses in 100 ms (i.e., 30 Hz) over the left hMT+. On each trial, the participants had to verbally report whether they perceived phosphenes or not and, if they positively reported phosphenes, whether these were stationary or exhibited some kind of moving or flickering patterns. For the stimulation over the left hMT+, the coil was always held tangential to the skull with the handle pointing upwards. This coil orientation has been shown to successfully produce interference with visual motion processing in previous studies [49,50,56,57]. The stimulation site was adjusted based on the characteristics of the phosphenes (e.g., moving, flickering, vivid, large), within 1 cm of radius from the point found with the craniometric procedure (i.e., 3 cm dorsal to inion and 5 cm leftward). Therefore, after the phosphene threshold phase, it is very likely that the stimulated area was hMT+ rather than other more posterior areas, such as V3B/KO or LOC. In general, in the present study the stimulation site was as in [50] (see Figure 2 of [50]).

All our participants reported the perception of either moving or flickering phosphene patterns during stimulation of left hMT+. The mean rTMS intensity over hMT+ was 53.5% (SD: 6.39%) and 56.9% (SD: 6.47%) for experiments 1 and 2, respectively. An independent t-test revealed no significant difference between the stimulation intensities used in the two experiments (Mann–Whitney U = 67.5, *p* = 0.268). In a separate session, but on the same day, we also stimulated Cz as a control site, to control for rTMS-related non-specific effects. The stimulation intensity over Cz was the same as for hMT+. At the beginning of each session, we estimated individually for each participant the phosphenes threshold delivering rTMS over hMT+, then the order of stimulation sites was randomized across participants. For the stimulation over Cz, the coil was always held tangential to the skull with the handle pointing backward. This stimulation regime is the same as used in the main experiment (see the Procedure section).

#### 2.1.5. Procedure

**Figure 1:**
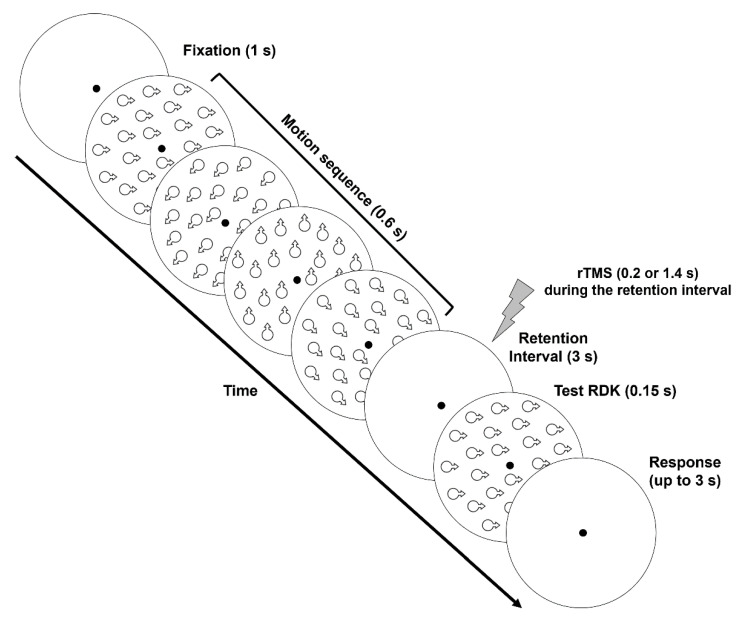
Schematic representation of stimuli and procedure used in the main rTMS VSTM experiment. An exemplary series of four RDKs moving in different directions is represented (sample motion sequence). After the 3 s retention interval, a test RDK is presented with the same direction as the first RDK in the sample motion sequence. rTMS (30 Hz) was delivered during the retention interval either 0.2 s or 1.4 s after the offset of the sample motion sequence. On each block, rTMS trials were randomly interleaved with No-TMS trials. Participants had 3 s to report whether the test RDK was presented or not in the sample motion sequence (Yes/No task).

The procedure used in the experiment consisted of two phases: (i) Training phase on motion direction discrimination. Participants were trained in a motion-direction discrimination task to make sure that they were able to discriminate the direction of moving stimuli [38,41]. This phase of the experiment consisted of a single presentation interval (duration 0.15 s) in which an RDK was displayed at the center of the screen. The motion sequence of the RDK was the same as reported in the Stimuli section. Participants had to discriminate the motion direction of the coherent RDK, which could move in one of the eight cardinal and intercardinal directions (8AFC). Observers reported the motion direction of the coherent RDK using one of eight designated keys of the keypad of a standard UK computer keyboard. Each block consisted of 64 trials (with each direction presented 8 times), and participants performed as many blocks as needed to get an accuracy ≥ 0.95. (ii) Main VSTM experiment. The procedure used in the main experiment was similar to that used by Stäblein et al. [58]. Each trial began with a fixation point presented for 1 s. The sample interval was composed of four RDKs (0.15 s each) presented in succession and with no blank interval between them. After the last RKD of the series, and after a retention interval of 3 s, another RDK was presented as a test stimulus (Figure 1). The test RDK had the same properties as the RDKs presented in the sample motion sequence. Participants were asked to memorize the direction of the four RDKs presented in the sample and report whether the direction of the test RDK was presented or not in the motion sequence (Yes/No task) by using the ‘K’ button to report ‘present’ or the ‘M’ button to report ‘absent’. When the direction of the test RDK was present in the motion sequence, this could be the same as the RDK direction in any position of the sample motion sequence. On the other hand, when the direction of the test RDK was not present in the motion sequence, its direction was randomly chosen from the four pre-defined directions not shown in the sample motion sequence. For the sake of simplicity, we will refer to target RDK indicating the RDK in the sample motion sequence having the same direction as that of the test RDK. Participants had 3 s to respond after the presentation of the test RDK. In each rTMS session, the test RDK direction was included in the sample sequence in half of the trials, with an equal probability of having the same direction either of the first, second, third, or fourth RDK in the sample motion sequence. In the other half of the trials, the test RDK had a different direction than those presented in the sample motion sequence.

For experiment 1, the temporal characteristics of the TMS stimulation were mainly based on the studies of [10,59]. In each session, rTMS (3 pulses during a 100 ms interval; 30 Hz) was delivered either 0.2 s (early rTMS) or 1.4 s (late rTMS) after the offset of the sample motion sequence and during the 3 s retention interval. This procedure was designed to induce a stimulation-related interference either during the encoding phase (early TMS) or on the retention phase (late rTMS) of the memory items, respectively. A rapid sequence of pulses at 30 Hz has been shown to improve the efficacy and reliability of the interference effects of TMS compared to lower stimulation frequencies [60]. rTMS trials were interleaved by trials with no stimulation (i.e., No-TMS trials). Each combination of target serial position in the sample (i.e., first, second, third, or fourth serial position), test RDK present or absent in the sample motion sequence, and TMS interval (i.e., No-TMS, early TMS, and late TMS) was presented six times. Therefore, each participant completed 144 trials (i.e., 4 target serial positions × 2 test RDKs [present/absent] × 3 TMS intervals × 6 repetitions) split into 8 blocks of 18 trials each. This was done to allow frequent breaks between blocks to avoid the cumulative effects of rTMS and limit fatigue. Different conditions were randomly presented within each block. Within each session, the stimulation site was kept the same. Before the main VSTM experiment participants were familiarized with the experimental procedure and completed a practice block of 24 trials (i.e., 4 target serial positions × 2 test RDK [present/absent] × 3 repetitions).

#### 2.1.6. Data Analysis

Individual hit rates (H) (i.e., when the participant correctly reported that the test RDK direction was present in the sample motion sequence) and individual false alarm rates (F) (i.e., when the participants erroneously reported that the test RDK direction was present in the sample motion sequence) were calculated as in [59]. H and F rates were then converted in non-parametric measures of sensitivity and bias; called *A* and *b*, respectively. The *A* index is the corrected version of the *A′* index proposed by [61] and the *A″* index proposed by [62], and it was calculated with the correction introduced by [63]. We used a non-parametric measure of sensitivity to deal with the presence of some H = 1 (13.5% out of the total hits values calculated (i.e., 21/156); there were no F = 0) and the small number of responses per condition (i.e., 6 repetitions per condition). *A* sensitivity values ranges from 0 to 1.0, with 0.5 being considered the chance level and 1.0 perfect performance. *b* values were log-transformed to get a symmetric bias measure with respect to zero. The non-parametric bias measure *log(b)* ranges from −1.0 (extreme bias in favor of yes responses) to 1.0 (extreme bias in favor of no responses). *A* value of 0.0 means no response bias [64]. Sensitivity/accuracy (*A* values) and bias (*log(b)*) values were analyzed using generalized linear mixed-effects models (GLMM) with ‘*lme4*’ package [65]. For more details on data analysis see the Supplementary Materials.

### 2.2. Results

#### 2.2.1. Main VSTM Experiment: Sensitivity (A)

**Figure 2:**
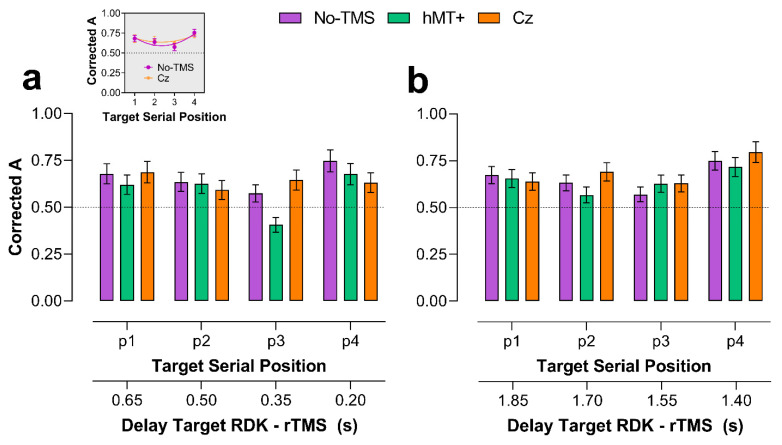
Mean *A* values for each serial position of the target RDK (1 to 4) and each TMS condition (i.e., No-TMS, and rTMS over hMT+ and Cz). The plotted data and error bars were estimates from the output of the selected models for early and late rTMS. The horizontal dashed lines indicate the chance level (*A* = 0.5). The secondary x-axes indicate the time delay (in seconds) between the offset of the target RDK in the motion sequence and the onset of the rTMS. (**a**) Mean *A* values estimated for early rTMS (i.e., 0.2 s after the offset of the motion sequence). In this case, for target in position 1, rTMS was delivered after 0.65 s from the offset of the first RDK. The insert above panel (a) shows the quadratic fit to the *A* values of the No-TMS and Cz conditions (averaged over the early and late rTMS conditions) of the form: $y=b0+b1x+b2x^{2}$, where b0, b1,b2 are the coefficients of the polynomial function. No-TMS: $b0=0.90,\;b1=-0.26,\;b2=0.06,\;R^{2}=0.8,\;SS=0.003$; Cz: $b0=0.80,\;b1=-0.14,\;b2=0.03,\;R^{2}=0.75,\;SS=0.0014$. (**b**) Mean *A* values estimated for late rTMS (i.e., 1.4 s after the offset of the motion sequence). Error bars ±SEM.

The results of the control experiment for motion direction discrimination are reported in the Supplementary Materials. Figure 2 reports *A* values for each stimulation condition and target serial positions. Data from early and late rTMS were analyzed separately to distinguish rTMS effects on the encoding and storing/retention phases of motion information.

#### 2.2.2. Early rTMS

Figure 2a shows *A* values for early rTMS. A Shapiro–Wilk test showed that residuals were not normally distributed (*W* = 0.97, *p* = 0.0013) with a negative skewness of −0.554 (SE = 0.192). Seven outlier data points were identified (i.e., *A* < 0.5) and included in the analysis. A Gamma function and *identity* link transformation function were used in the GLMM model. In the analysis, we included *A* values estimated in all the stimulation conditions (i.e., No-TMS, rTMS delivered over hMT+ and Cz). For the No-TMS trials, we calculated the average between the No-TMS trials in the hMT+ condition and those in the Cz condition. Additionally, we use the same No-TMS *A* values for the early and late rTMS conditions.

The selected model included as fixed factors the stimulation condition (i.e., No-TMS, rTMS over hMT+ and Cz), target position in the motion sequence and the interaction between stimulation condition and target position. Random effects of the selected model included random intercepts across participants and the participants’ random slopes for the stimulation condition. The selected model reported a significant fixed effect of the stimulation condition (*χ^2^* = 6.24, *df* = 2, *p* = 0.044), target position (*χ^2^* = 44.78, *df* = 3, *p* < 0.0001) and a significant interaction between stimulation condition and target position (*χ^2^* = 18.63, *df* = 6, *p* = 0.0048).

For the stimulation condition, pairwise post hoc comparisons, corrected with false discovery rate (FDR; α = 0.05) [66], did not reveal any significant difference between the three stimulation conditions (No-TMS, rTMS over hMT+ and Cz) (all *adjusted-p* > 0.05).

For the target position, pairwise post hoc with FDR correction revealed a significant difference between positions 1 and 3 (*adjusted-p* = 0.0026) and between positions 3 and 4 (*adjusted-p* = 0.0006).

For the stimulation condition x target RDK position interaction, pairwise post hoc comparisons with FDR correction revealed a significant difference between target position 1 and 3 when rTMS was delivered over hMT+ (*adjusted-p* < 0.001), between target position 2 and 3 when rTMS was delivered over hMT+ (*adjusted-p* < 0.001), between target position 3 and 4 when rTMS was delivered over hMT+ (*adjusted-p* < 0.001), between rTMS over hMT+ and Cz for target position 3 (*adjusted-p* = 0.0005), and between No-TMS and hMT+ for target RDK in position 3 (*adjusted-p* = 0.0185) (for the interaction, the FDR correction was applied for 66 tests). Additionally, for target RDK position 3, there was not a significant difference between No-TMS and Cz conditions (*adjusted-p* = 0.764).

Overall, the results for early rTMS during the retention interval show low sensitivity values across all the conditions, suggesting that the task was quite difficult. Sensitivity was lower when the target was presented in the second and third serial positions, suggesting the presence of serial effects in motion direction recalling (i.e., primacy and recency effects [67,68]. This was evident especially for the No-TMS condition for which a trend analysis reported a significant quadratic trend (*F*_1,12_ = 10.81, *p* = 0.006) but not a linear trend (*F*_1,12_ = 0.91, *p* = 0.36), and for the Cz condition, when averaging early and late rTMS data (*F*_1,12_ = 7.83, *p* = 0.016). For the No-TMS condition, the minimum value of the quadratic function was *A* = 0.591, corresponding to a target serial position of 2.35, whereas, for the Cz condition, the minimum value of the quadratic function was *A* = 0.64, corresponding to a target serial position of 2.32. On the other hand, the quadratic trend was not evident for the hMT+ condition (*F*_1,12_ = 2.07, *p* = 0.18). rTMS over hMT+ further reduced the sensitivity for the target when it was delivered 0.35 s after the offset of the third RDK in the motion sequence. This also suggests that rTMS maximally interfered with the encoding of the moving stimuli when delivered 0.35 s after the presentation of the moving RDK (Figure 2a).

A series of one-sided one-sample permutation tests (sampling permutation distribution 5k) were performed for each condition on *A* values to assess whether accuracy/sensitivity values across the stimulation conditions were greater than the chance level (0.5). The results showed that for the No-TMS condition all the *A* values were significantly greater than 0.5 (*p* < 0.01), but the *A* value estimated in position 3 (*p* = 0.0634). For the hMT+ condition we found the same results, with all the *A* values significantly higher than the chance level (*p* < 0.05) but the value estimated in position 3 (*p* = 0.41). For the Cz condition, all the *A* values were significantly higher than 0.5 (all *p* < 0.01).

#### 2.2.3. Late rTMS

For late rTMS (Figure 2b), *A* values were also analyzed using GLMMs. A Shapiro–Wilk test showed that residuals were not normally distributed (W = 0.973, *p* = 0.0039) with a negative skewness of −0.565 (SE = 0.192). Seven outliers (low A values) were identified and included in the analysis. For A values estimated in the late rTMS condition the selected model included as fixed factors the stimulation condition (i.e., No-TMS, rTMS over hMT+ and Cz), target position and the interaction between stimulation condition and target position. Random effects of the selected model included random intercepts across subjects and the participants’ random slopes for the stimulation condition. As for early rTMS, the model included a Gamma function and an *identity* link function. The model did report a significant effect of the target position (χ^2^ = 20.43, df = 3, *p* = 0.00014), but not a significant effect of the stimulation condition (χ^2^ = 1.25, df = 2, *p* = 0.54) or a stimulation condition × target position interaction (χ^2^ = 6.29, df = 6, *p* = 0.39). For the target position, pairwise post hoc with FDR correction revealed a significant difference between position 1 and 4 (*adjusted-p* = 0.011), position 2 and 4 (*adjusted-p* = 0.001) and position 3 and 4 (*adjusted-p* = 0.0001).

rTMS delivered approximately in the middle of the retention interval did not interfere with the storing/retention of the motion information. As for the early rTMS condition, we performed a series of one-sided one-sample permutation tests. For the hMT+ condition we found that all the *A* values were significantly higher than the chance level (*p* < 0.05), but the mean *A* value estimated in position 2 (*p* = 0.055). For the Cz condition, all the *A* values were significantly higher than 0.5 (all *p* < 0.01).

#### 2.2.4. Main VSTM Experiment: Bias

##### Early rTMS

**Figure 3:**
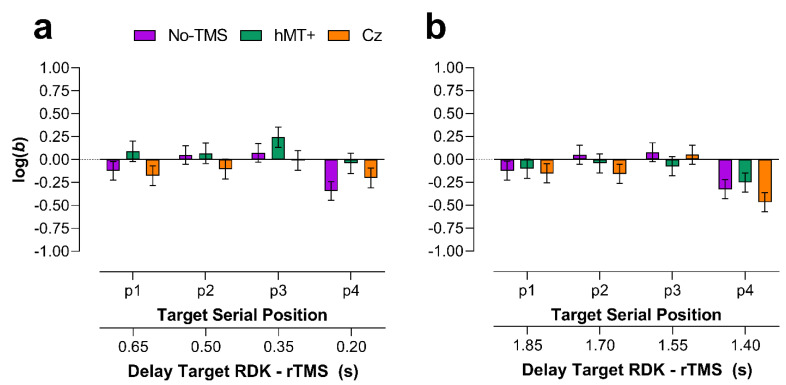
Mean bias [*log*(*b*)] for each serial position of the target RDK (1 to 4) and each TMS condition. The plotted data and error bars were estimates from the output of the selected and fitted GLMM models for early and late rTMS. Data are symmetrical with respect to zero, with negative values indicating a bias towards *yes* responses, and positive values towards *no* responses. The secondary x-axes indicate the time delay (in seconds) between the offset of each RDK in the motion sequence and the onset of the TMS. (**a**) *log*(*b*) values estimated for early rTMS. (**b**) *log*(*b*) values estimated for late rTMS. Error bars ±SEM.

Figure 3 shows *log(b)* values for each stimulation condition and target position. As for sensitivity values, data from early and late rTMS were analyzed separately. For early rTMS (Figure 3a), data were analyzed using GLMMs. A Shapiro–Wilk test on non-transformed *b* values showed that residuals were not normally distributed (*W* = 0.978, *p* = 0.013) with a positive skewness of 0.112 (SE = 0.192). Six outliers (positive and high *b* values) were identified and included in the analysis. In the analysis, we included *b* values estimated in all the stimulation conditions (i.e., No-TMS, and rTMS delivered over hMT+ and Cz). As for *A* values, for No-TMS trials, we took the average between the No-TMS trials in the hMT+ condition and those in the Cz condition.

The selected model included as fixed factors the stimulation condition (i.e., No-TMS, rTMS over hMT+ and Cz), target position and the interaction between stimulation condition and target position. Random effects of the selected model included random intercepts across participants and the participants’ random slopes for stimulation condition. The GLMM included a Gamma function and a *log* link function, so that *b* values were log-transformed. The model reported only a significant fixed effect of the target position (*χ^2^* = 15.16, *df* = 3, *p* = 0.0017), but not of the stimulation condition (*χ^2^* = 5.17, *df* = 2, *p* = 0.076), and stimulation condition x target position interaction (*χ^2^* = 3.5, *df* = 6, *p* = 0.74).

FDR corrected post hoc comparisons for the target position reported a significant difference between positions 2 and 4 (*adjusted-p* = 0.034) and between positions 3 and 4 (*adjusted-p* = 0.001) (FDR was applied for 6 comparisons).

A series of two-sided one-sample permutation tests (sampling permutation distribution 5k) were performed for each condition on *log(b)* values to assess whether the bias measures were significantly different from zero. The results showed that for the No-TMS condition all the log(b) values were not significantly different from zero (*p >* 0.05) but the *log(b)* value in position 4 (*p* = 0.01). For the hMT+ and Cz conditions, all the *log(b)* values were not significantly different from zero (*p >* 0.05), indicating no response bias across the conditions.

##### Late rTMS

Figure 3b shows *log(b)* values for the late rTMS. A Shapiro–Wilk test on non-transformed *b* values, showed that residuals were not normally distributed (*W* = 0.981, *p* = 0.03) with a positive skewness of 0.516 (SE = 0.192). Four outliers (positive and high *b* values) were identified and included in the analysis. In the analysis, we included *b* values estimated in all the stimulation conditions. The selected model included as fixed factors the stimulation condition (i.e., No-TMS, rTMS over hMT+ and Cz), target position, and the interaction between stimulation condition and target position. Random effects of the selected model included only random intercepts across participants. The model included a Gamma function and a *log* link function. The selected model reported only a significant fixed effect of the target position (*χ^2^* = 22.5, *df* = 3, *p* < 0.0001), but not of the stimulation condition (*χ^2^* = 2.04, *df* = 2, *p* = 0.36), and stimulation condition × target position interaction (*χ^2^* = 3.89, *df* = 6, *p* = 0.69). FDR corrected post hoc comparisons for the target position reported a significant difference between positions 1 and position 4 (*adjusted-p* = 0.014), between position 2 and position 4 (*adjusted-p* = 0.0009) and between positions 3 and 4 (*adjusted-p* < 0.0001).

A series of two-sided one-sample permutation tests (sampling permutation distribution 5k) were performed for each condition on *log(b)* values to assess whether the bias measures were significantly different from zero. The results showed that for the hMT+ and Cz conditions, only the bias estimated for target RDKs in position 4 was significantly different from zero (*p* = 0.038 and *p* < 0.0001, for hMT+ and Cz respectively). For these two conditions, the bias was negative thus indicating more ‘yes/present’ responses.

### 2.3. Discussion

The results of experiment 1 showed that when rTMS was delivered over the left hMT+ interfered with the encoding phase of the third target RDK in the motion sequence, that is when rTMS was delivered after 0.35 s from the offset of the target RDK (or 0.5 s after its onset). Though the serial position was not crucial for experiment 1, this effect is specific for the serial position in the motion sequence as there was not a significant difference between No-TMS and Cz for the same target serial position. However, the decrement in sensitivity/accuracy obtained after stimulating hMT+ was significantly lower than the accuracy values estimated in the No-TMS and Cz conditions. This effect is similar to that reported by van de Ven et al. [59], in which rTMS interfered with the encoding of the short-term representation of a complex shape when delivered 0.2 s after the stimulus presentation. It should be noted the difference between our results and those of van de Ven et al. [59] in terms of TMS-induced interference with encoding. Though this difference could depend on the stimuli used and their timing, and the task [69], there is evidence that encoding in VSTM can take up to 0.6 s. Fukuda and Vogel [70] manipulated the stimulus-to-mask interstimulus interval (ISI) to investigate the duration of the encoding phase. The memory sample consisted of three pictures of real objects presented simultaneously for 0.15 s, after that a 0.05 s mask was presented at each object’s location at variable sample-to-mask ISI (i.e., 0, 0.1, 0.3, 0.6, and 1.5 s). Finally, a test stimulus was presented and consisted either of one of the old objects shown in the memory sample or a new object. Participants were asked to indicate whether the test object was present or not in the memory sample. The results showed that masking was effective in disrupting encoding up to 0.6 s from the stimulus onset, that is within our temporal window of TMS-induced interference with encoding for the sample motion sequence.

For the late TMS condition we found only an effect of the target position, but no effect of the TMS. In both early and late TMS conditions, the RDK in the last serial position (i.e., fourth position) had always higher sensitivity/accuracy than the other positions, suggesting a recency effect resistant to TMS-induced interference. Despite no main effect of rTMS and no interaction with the target position, one-sided one-sample permutation tests found that rTMS over hMT+ produced *A* values that were not different from chance level only when the target was in the second serial position. This result suggests an effect of rTMS specific for intermediate target positions, similarly to what was found in the early rTMS condition. Additionally, for the response bias, we found only a significant effect of the target position, with less conservative responses (i.e., more ‘yes/present’ responses) when the target was presented in the fourth serial position. However, no significant effects of the stimulation were found, suggesting that rTMS did not introduce any specific response bias.

## 3. Experiment 2

### 3.1. Methods

In the second experiment we assessed the effects of rTMS on the precision of VSTM for motion direction Importantly, we employed only the early rTMS condition, as in experiment 1 we found an effect of the stimulation only for the early condition. We used the same memory load as in experiment 1. Two of the authors (AP, FG) and six naïve participants took part to experiment 2. Apparatus, stimuli, and brain stimulation regime were the same as in experiment 1. However, in experiment 2 the RDKs could drift in a range of directions between 0 and 359.9 deg. To assign a specific motion direction to each RDK in the stimulus series, we originated an array of 3.6k directions ranging from 0 deg to 359.9 deg in steps of 0.1 deg. For each motion sequence, we pseudo-randomly chose four directions with the constraints that the minimum pairwise angular separation between two consecutive directions was 45 deg, and that all the motion directions in the sequence were different.

The procedure used in experiment 2 consisted again of two phases: (i) An initial control experiment in which participants were trained in a motion-direction discrimination task to match their initial performance in terms of motion direction discrimination [38,41]. This initial control experiment was the same as that used in experiment 1. (ii) Main VSTM precision experiment. The procedure used in the main experiment was similar to that used by Zokaei et al. [31] in their first experiment. The sample interval was composed of a series of four RDKs with a duration of 0.5 s each. The duration of each RDK was longer than that used in the previous experiments as participants were asked to remember both the directions of the four patches and their position in the motion sequence.

**Figure 4:**
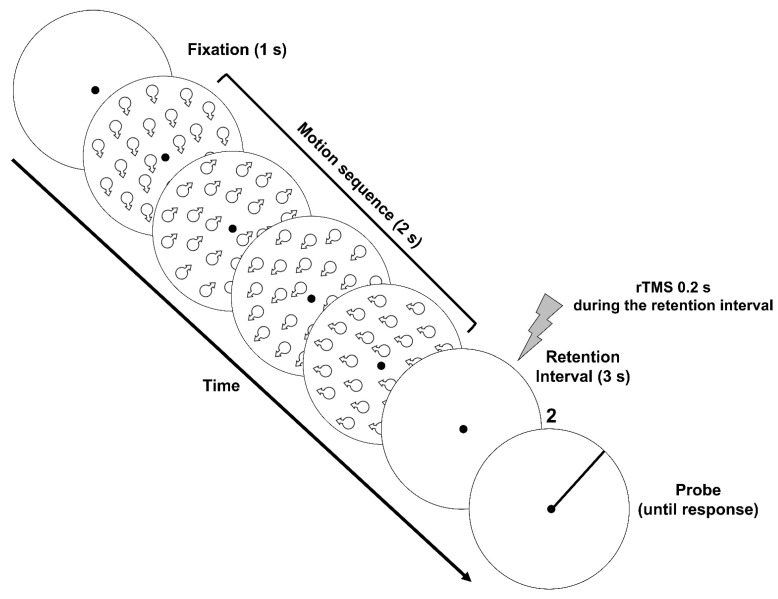
Schematic representation of stimuli and procedure used in experiment 2. An exemplary series of four moving RDKs is represented and the target to report is the second moving RDK in the series (indicated by the digit “2” above the probe stimulus). The small arrows in the motion sequence indicate given stimulus directions in a typical trial. For the sake of illustration, they depict motion direction in the figure but were never presented during the actual experiment. The motion sequence was composed of a series of four RDKs presented for 0.5 s each (2 s in total).

The length of the stimulus series was always composed of four moving RDKs. After the last RKD of the series, and after a blank interval of 3.0 s, a probe was presented. The probe consisted of a frame circle of approximately the same diameter as the circular aperture of the RKDs, with a line of the same length of the radius starting from the center (Figure 4). At the top of the circular frame a digit indicated the target RDK in the stimulus series, the direction was to be reported (e.g., “2” = report the second RDK direction, “3” = report the third RDK direction, etc.). Participants were asked to adjust the orientation of the line inside the circular frame by using either the left or the right arrow, to match the direction of motion of the target RDK indicated by the digit appearing above the frame circle. The probe line was randomly positioned on a trial-by-trial basis around the circumference. The probability of probing any of the RDKs within the sequence was kept constant for all items in the series. The probe display was presented until participants had reported the direction of the target RDK and pressed the space bar to continue with the subsequent trial. Participants were instructed to respond as accurately as possible with no time pressure (reaction times were not recorded).

In total, there were 120 trials for each stimulation site (hMT+ and Cz). The total amount of trials was divided into 8 blocks of 15 trials each. Before the main VSTM precision experiment, participants were familiarized with the experimental procedure and completed practice blocks of 12 trials, each with no TMS in which the target serial position was randomized across trials (i.e., 3 trials per each spatial position). Participants performed up to three training blocks to familiarize themselves with stimuli and the task. The training blocks were also analyzed to assess whether participants performed the VSTM precision task above chance. During the main experiment, rTMS (30 Hz) was delivered on 50% of the trials 0.2 s after the onset of the 3 s retention interval.

Finally, errors between the target RDK direction and participants’ responses were fitted with the variable precision (VP) model [33,71,72], to assess whether components of visual short-term memory differed across the three stimulation conditions (No-TMS, hMT+, and Cz).

### 3.2. Precision Calculation

Precision was defined as the inverse of the circular standard deviation of the angular distance (error in radians) between the target direction and the participant’s response. See the Supplementary Materials for more details on precision calculation. 

### 3.3. Results

#### 3.3.1. Precision of VSTM for Motion Direction

**Figure 5:**
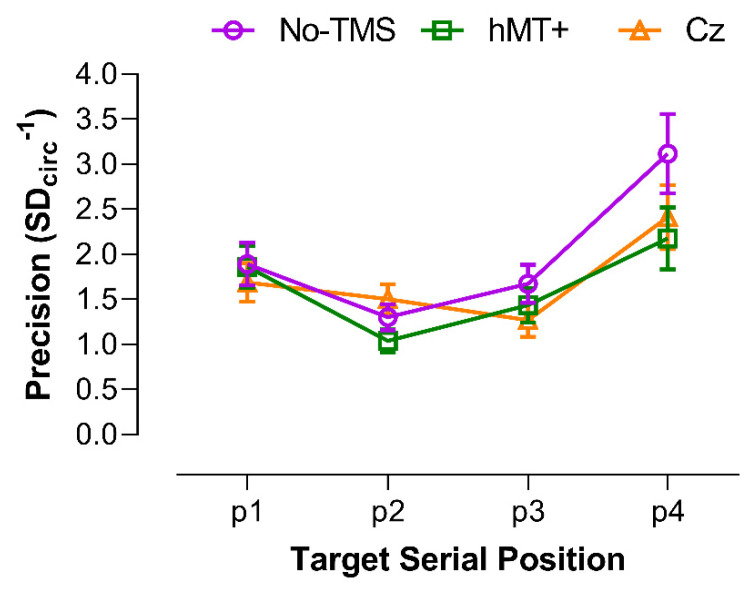
VSTM precision (calculated as the inverse of the circular standard deviation of the angular distance [error in radians] between the target direction and the participant’s response) for each stimulation type (different curves) as a function of the target serial position in the motion sequence. Error bars ±SEM.

The results of the control experiment for motion direction discrimination are reported in the Supplementary Materials. Figure 5 shows VSTM precision as a function of the target serial position for each stimulation condition. As in the previous experiments, precision data were analyzed using GLMMs. A Shapiro–Wilk test showed that residuals were not normally distributed (*W* = 0.82, *p* < 0.001) with a positive skewness of 1.456 (SE = 0.242). Eleven outliers were identified and included in the analysis. The selected model included as fixed factors the stimulation condition (i.e., No-TMS, rTMS over hMT+ and Cz), target position in the motion sequence and the interaction between stimulation condition and target position. Random effects of the selected model included random intercepts across participants and the participants’ random slopes for the target position. The model included an Inverse Gaussian distribution and an *identity* link function. The selected model reported a significant fixed effect of the stimulation condition (*χ^2^* = 11.5, *df* = 2, *p* = 0.0032), a significant effect of target position (*χ^2^* = 34.91, *df* = 3, *p* < 0.0001), and a significant interaction between stimulation condition and target position (*χ^2^* = 19.15, *df* = 6, *p* = 0.0039). 

For the stimulation condition, pairwise post hoc comparisons with FDR correction, revealed a significant difference between Cz and No-TMS (*adjusted-p* = 0.025), between hMT+ and No-TMS (*adjusted-p* = 0.0032), but not between hMT+ and Cz (*adjusted-p* = 0.27). 

For the target position, pairwise post hoc with FDR correction, revealed a significant difference between position 1 and 2 (*adjusted-p* = 0.0004), between position 1 and 3 (*adjusted-p* = 0.0032), between position 1 and 4 (*adjusted-p* = 0.0098), between position 2 and 4 (*adjusted-p* = 0.0001), between position 3 and 4 (*adjusted-p* = 0.004), but not between position 2 and 3 (*adjusted-p* = 0.24). This suggests the presence serial effects in remembering the target motion direction in the motion sequence, with positions 2 and 3 showing lower precision than positions 1 and 4. A trend analysis on precision values showed a significant linear (*F*_1,7_ = 6.79, *p* = 0.035) and quadratic (*F*_1,7_ = 13.16, *p* = 0.008) trend for the No-TMS condition, only a significant quadratic trend for the hMT+ condition (*F*_1,7_ = 13.65, *p* = 0.008) (linear: *F*_1,7_ = 0.97, *p* = 0.36), but any significant trend for the Cz condition (linear: *F*_1,7_ = 4.95, *p* = 0.061; quadratic: *F*_1,7_ = 4.87, *p* = 0.063).

For the stimulation condition x target position interaction, selected pairwise post hoc comparisons with FDR correction are shown in Table 1. The main result is that when the target RDK was in position 2 (i.e., when rTMS was delivered 1.2 s after the offset of the target patch in position 2), rTMS over hMT+ negatively affected the VSTM precision with respect to both Cz and No-TMS. Additionally, for this target position, there was not a significant difference between Cz and No-TMS.

A series of one-sided one-sample permutation tests (sampling permutation distribution 5k) were performed for each condition to assess whether precision values across the stimulation conditions and target positions were significantly higher than zero (i.e., chance level). The results showed that all the precision values were significantly higher than zero (all *p* < 0.01).

#### 3.3.2. Modeling of Visual Short-Term Memory

Delayed estimation tasks are particularly useful to assess the components of VSTM. In this account, in the variable precision (VP), model precision is variable across items and trials and previous studies have shown that visual short-term memory precision is indeed continuous and variable across memory items and trials [27,33,71,72]. In the VP model, the amount of resource an item receives, thus regulating its encoding precision, varies randomly across memory items and trials and decreases with set size. The VP model we fitted to error values was characterized by three parameters: guess rate (g), the mean standard deviation of responses (meanSD) and the standard deviation of response error (SDvar). The guess rate (g) expresses the probability with which the observer does not remember the direction of the target patch probed in the test phase and consequently guesses randomly. MeanSD represents the mean standard deviation of the precision of the remembered items, and it is inversely related to precision; high values in meanSD indicate a less precise memory representation. SDvar indicates intertrial variation in memory precision; high values of SDvar indicate high trial-to-trial variability. See the Supplementary Materials for more details on the VP model and the fitting procedure.

#### 3.3.3. Results of the Variable Precision Model

**Figure 6:**
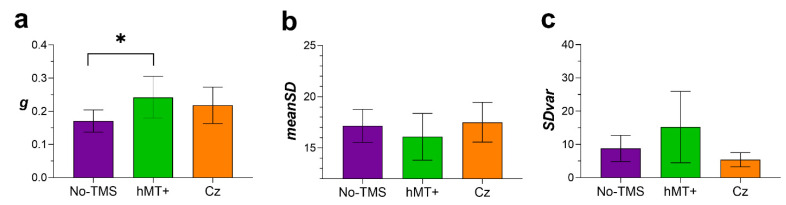
Mean parameters’ estimates of the variable precision model. (**a**) *g* (random guessing) for No-TMS, hMT+ and Cz. (**b**) *MeanSD* (mean standard deviation of precision) for the three stimulation conditions. (c) *SDvar* (intertrial variation in memory precision). The asterisk represents the significant difference between No-TMS and hMT+ for *g*. Error bars ±SEM.

Figure 6 shows the estimated parameters of the VP model. A Shapiro–Wilk test for normality revealed that residuals for g and SDvar were not normally distributed (g: W = 0.9, *p* = 0.022; meanSD: W = 0.96, *p* = 0.52; SDvar: W = 0.52, *p* < 0.0001); therefore, the estimated parameters were analyzed using the aligned rank transform for nonparametric factorial analyses [73]. This analysis implements pre-processing steps that align not normally distributed data before applying averaged ranks, after which ANOVA or a linear mixed model can be performed. This analysis was performed by using the statistical software R and the “*ARTtool*” package (http://depts.washington.edu/madlab/proj/art/). After the rank assignment, we performed a linear mixed model using the lme4 package for R [65] with stimulation type as within-subjects factors, and with random intercept across subjects.

For g (Figure 6a), the non-parametric factorial analysis revealed a significant effect of the stimulation (F_2, 14_ = 4.67, *p* = 0.028). Post-hoc comparisons corrected for the false discovery rate (FDR; α = 0.05) revealed only a significant difference between No-TMS and hMT+ (*p* = 0.03). For meanSD (Figure 6b) and SDvar (Figure 6c), the non-parametric factorial analysis did not report a significant effect of the stimulation type (F_2, 14_ = 0.11, *p* = 0.89; F_2, 14_ = 0.195, *p* = 0.82, for meanSD and SDvar, respectively).

In sum, fitting the errors data with the VP model [71] revealed that the three stimulation types do not differ in terms of the mean standard deviation of the precision of the remembered items (meanSD) or in terms of trial-to-trial precision variability (SDvar). However, rTMS over hMT+ significantly increased guess rate (g) with respect to the No-TMS condition, though the probability of guessing rate in the hMT+ condition was not significantly higher than the guess probability in the Cz condition.

## 4. Discussion

The results of experiment 2 show that rTMS delivered over hMT+ 0.2 s after the motion sequence in the retention interval mainly interferes with the precision of the target patch in position 2. This suggests the presence of serial effects, with positions 1 and 4 showing the highest VSTM precision (i.e., primacy and recency effects) and positions 2 and 3 the lower precision, though there were no evident rTMS effects for position 3. A variable precision (VP) model fitted on response errors showed that these results may depend on a higher rate of random responses when rTMS was delivered over hMT+, with respect to the No-TMS condition, though the guess rate estimated in the Cz condition was approximately the same.

## 5. General Discussion

In two experiments, we investigated the neural underpinnings of VSTM for a sequence of moving stimuli by interfering with the encoding and retention of the memory trace by delivering rTMS over hMT+, a critical area for motion processing [40,74,75,76,77,78,79]. In the first experiment, we used a recognition task in which participants were asked to indicate whether a test RDK had the same motion direction as any of the four RDKs shown in the memory sample. rTMS was delivered either on the early or the late part of a 3 s retention interval. The results showed that, when rTMS was delivered at the early stage of the retention interval (i.e., 0.2 s) over hMT+, the stimulation interfered with participants’ sensitivity when the test RDK corresponded to the third RDK presented in the temporal sequence. On the other hand, early TMS did not influence the bias measure.

Furthermore, these results showed that early stimulation-related interference over the hMT+ in the retention interval might interfere with the encoding of the entire motion sequence and the formation of the memory trace of the four motion directions. On the other hand, stimulation of the hMT+ later in the retention interval did not induce any significant modulation of sensitivity or bias. Therefore, we could argue that the lack of late TMS-induced interference during the retention interval suggests that hMT+ may not be directly involved in the retention process.

Importantly, these findings suggest that the visual complex hMT+ seems to be causally involved in the encoding of a sequence of visual moving stimuli in VSTM, whereas its role in retaining the motion sequence remains unclear. There is brain imaging evidence that multiple cortical sites may contribute to the retention and precision of visual information in VSTM. For example, the superior intraparietal sulcus (sIPS) may be involved in the modulation of the variability of visual working memory precision under increased memory load [80]. Additionally, a recent study by Zhao et al. [81] showed that the lateral occipital cortex (LOC) supports visual working memory precision, while the communication between the inferotemporal junction and LOC is modulated by the memory load, suggesting the presence of distinct neural mechanisms encoding for the memory load and precision variability.

In the second experiment, the precision of the encoded information was tested by asking participants to report the motion direction of one of the four RDKs presented in the memory sample with the adjustment method. The results showed that not only the functional integrity of hMT+ is causally involved in the strength of representation of items stored in VSTM, as measured with a sensitivity index, but it is also involved in encoding the precision of the memorized items. In fact, in experiment 2 we found a disruption of precision performance when rTMS was delivered over hMT+ and only for RDKs in the second temporal position.

Interestingly, previous studies have found that rTMS over the hMT+ showed different modulatory effects on the memory trace of stimuli presented in a temporal serial sequence. For example, Zokaei et al. [82], using a temporal sequence of two RDKs, found that rTMS over the hMT+ had opposite effects on memory precision depending on the position of the item in the temporal sequence. Specifically, in their study, rTMS delivered either after the first or second RDK decreased recall precision of motion direction only for the last item presented but improved the recall precision of the first item in the motion sequence. The authors suggested that rTMS may have interfered only with the encoding of the last item that was in a privileged activation state by virtue of recency. It could be that the smaller recency effect boosted the recall precision of the first item in the temporal sequence. However, our results showed decreased VSTM precision only for RDKs presented in the middle of the temporal sequence (i.e., second and third positions), but no significant modulation for the last privileged item of the sequence. However, this might depend on differences between the experimental designs and stimulation protocols.

Furthermore, the results of the variable precision (VP) model showed that, when rTMS was delivered over the hMT+, the probability of random responses significantly increased with respect to the No-TMS condition, but not with respect to Cz. We did not find a significant difference between No-TMS and Cz and between hMT+ and Cz for the guessing rate. The other parameters of the VP model were not affected by the stimulation. Increased guessing responses can result from forgetting, lapses of attention, or encoding failures. Since early TMS pulses were presented at the end of the encoding phase in the retention interval (i.e., 0.2 s after the offset of the motion sequence) [8,9,10,59], it is possible that rTMS increased random guesses by interfering with the encoding of the four stimuli in the temporal sequence [83]. However, given that there was not a significant difference between hMT+ and Cz in terms of guess rate, it is possible that the results observed might depend on rTMS-induced distractibility, though there was not a significant difference between No-TMS and CZ, if this was the case. rTMS-induced increase of guessing rate probability has also been shown in previous findings. Rademaker et al. [83], showed that when participants had to remember the orientations of four briefly presented Gabor patches, recall errors were smaller when the visual field location targeted by rTMS overlapped with that of the cued memory item, compared to errors for stimuli probed diagonally to rTMS. Furthermore, early TMS pulses (i.e., immediately after the offset of stimulus presentation) impaired performance at all four locations, compared to late pulses (delivered in the middle of a 2 s retention interval). A mixture model [84] fitted to response errors showed that VSTM for orientation was more precise for items proximal to the pulse location, irrespective of pulse timing. However, the guessing rate was higher with early TMS pulses than late pulses, regardless of stimulus location. The authors concluded that rTMS administered at the offset of the stimulus might disrupt early-phase consolidation of visual information. Taken together, these findings demonstrate the importance of the encoding phase mechanisms in early sensory cortices involved in the formation of short-term memory traces of visual information [59].

## 6. Limitations

One limitation of the present study is the lack of neuronavigation to identify the hMT+ area. Although the craniometric coordinates used in this study have been shown to be reasonably close to hMT+ coordinates defined using a combination of brain imaging and neuronavigation [50,85], the procedure could still entail a small error in locating the desired stimulation site. Another limitation of the study is the limited number of TMS time points used after the offset of the motion sequence, especially for experiment 1. This choice was based on our previous behavioral findings using visual masking after the presentation of the motion memory sample [10] and previous rTMS studies which used only two or three TMS time points during the retention interval to interfere with encoding and retention of the memory sample [59,83]. We acknowledge that a better characterization of the encoding time window for motion signals would require rTMS delivered over a range of time points from the offset of the memory sample (i.e., 0 s) up to the boundary of the encoding window for visual information (e.g., 0.6 s) [70].

## 7. Conclusions

Overall, these findings provide further evidence that items in VSTM are not all-or-none representations; instead, such representations have different degrees of resolution, depending on the allocation of memory resources, interference from other memory items, or from external factors such as perturbations induced by TMS. These types of representations are more compatible with a dynamic resource model of VSTM than with a limited capacity memory model [27,28,29,30,32,33]. In both experiments, primacy and recency effects were evident, hence performance was better for the first and last RDK stimulus of the motion sequence, and worse for the stimuli presented in the middle of the temporal sequence. We speculate that this effect of serial position might be due, rather than to the intervention of long- and short-term memory mechanisms, to the interference of items with adjacent serial positions [86]. When the item is in the first position of the sequence, (backward) interference will come only from the successive item; similarly, when the item is in the last position of the sequence, (forward) interference will come only from the previous item. However, when the item is in a middle position, interference will come from both the previous and successive items in the sequence, thus producing larger interference. The presence of this serial dependence effect, not only in an all-or-none recognition task but also in a more subtle motion direction recall, suggests that each item in VSTM has a different degree of strength or activation, thus leading to a different precision judgment. This, in turn, is in support of a model of VSTM with a variable resolution of each stored item, consistent with the assigned amount of memory resources or serial effects.

## Figures and Tables

**Table 1:** Selected post hoc comparisons between precision values for the different stimulation types and for each target position. Significant *p* values are indicated with an asterisk.

	Target Serial Position			
Stimulation Type Comparison	P1	P2	P3	P4
hMT+ vs. No-TMS	0.894	0.042 *	0.189	0.015 *
Cz vs. No-TMS	0.338	0.288	0.015 *	0.084
Cz vs. hMT+	0.375	0.006 *	0.204	0.287

## Data Availability

The data presented in this study are available on request from the corresponding authors.

## Supplementary Materials

The following are available online at https://www.mdpi.com/article/10.3390/brainsci11111471/s1.
