# Supplementary material for: Visual Short-Term Memory for Coherent and Sequential Motion: A rTMS Investigation
Source: Brain Sci. 2021 Nov 6;11(11):1471. doi: 10.3390/brainsci11111471 (PMC8615668; doi:10.3390/brainsci11111471)
Supplement: Supplementary file 1 [file brainsci-11-01471-s001.zip › brainsci-1377836-supplementary.pdf]

brain sciences

Supplementary Materials

Data Analysis

Individual Hit rates (H), i.e., when the participant correctly reported that the test RDK direction was present in the sample motion sequence, and individual False Alarm rates (F), i.e., when the participants erroneously reported that the test RDK direction was present in the sample motion sequence, were calculated as follows [1]:

$$H = \frac{\text{hits}}{\text{hits + misses}} \qquad \text{Eq.1}$$

$$F = \frac{\text{False Alarms}}{\text{False Alarms + Correct Rejections}} \qquad \text{Eq.2}$$

H and F rates were then converted in non-parametric measures of sensitivity and bias; called A and b, respectively. The A index is the corrected version of the A' index proposed by [2] and the A'' index proposed by [3], and it was calculated with the correction introduced by [4]. We used a non-parametric measure of sensitivity to deal with the presence of some $H = 1$ (13.5% out of the total *hits* values calculated, i.e., 21/156; there were no $F = 0$) and the small number of responses per condition (i.e., 6 repetitions per condition). A is a non-parametric estimate of the area under a proper Receiver Operating Characteristic (ROC) curve[1] [2] that passes through any single point $p = (F, H)$ and that is less sensitive to extreme hit and false-alarm rates than the classic *d-prime* [5]. As pointed out in [4,6], based on standard Signal Detection Theory (SDT), H and F rates are transformed into indices of sensitivity (i.e., *d-prime*) and bias based on the assumption of normality of signal and noise distributions with equal variance. However, other transformations are possible including those not tied to an underlying statistical detection model, including ones based on the range of proper ROC curves that could pass through any single point. Additionally, these assumptions cannot be tested in Yes/No tasks, as rating tasks are required for this purpose [6]. Therefore, we used A and b indexes as reported in [4]:

$$A = \begin{cases} \frac{3}{4} + \frac{H-F}{4} - F(1-\mathrm{H}) & \text{if } F \le 0.5 \le H; \\ \frac{3}{4} + \frac{H-F}{4} - \frac{F}{4H} & \text{if } F \le H < 0.5; \\ \frac{3}{4} + \frac{H-F}{4} - \frac{1-H}{4(1-\mathrm{F})} & \text{if } 0.5 < F \le H; \end{cases} \qquad \text{Eq.3}$$

$$b = \begin{cases} \frac{5-4H}{1+4F} & \text{if } F \le 0.5 \le H; \\ \frac{H^2+H}{H^2+F} & \text{if } F < H < 0.5; \\ \frac{(1-F)^2+(1-H)}{(1-F)^2+(1-F)} & \text{if } 0.5 < F < H; \end{cases} \qquad \text{Eq.4}$$

[1] Based on Zhang and Mueller [4], a proper ROC curve is a monotonically non-decreasing function with a non-increasing slope connecting the points (0,0) and (1,1) and necessarily lying above the line H = F. Therefore, a ROC curve between (0,0) and (1,1) is convex.

A sensitivity values ranges from 0 to 1.0, with 0.5 being considered the chance level and 1.0 perfect performance. Low values of *A* (i.e., below 0.5 and close to zero) could depend on sampling errors or response confusion [6]. *b* represents the slope of the proper ROC curve, and *b* values were *log*-transformed to get a symmetric bias measure with respect to zero. The non-parametric bias measure *log*(*b*) ranges from -1.0 (extreme bias in favor of *yes* responses) to 1.0 (extreme bias in favor of *no* responses). A value of 0.0 means no response bias [6]. *A* and *log*(*b*) values were the input data for the statistical analyses. Data were analyzed using R (R Core Team, 2019, v4.0.4; https://www.r-project.org/) in RStudio (RStudio Team, 2015, v1.4.1103; https://www.rstudio.com/).

Sensitivity/accuracy (*A* values) and bias (*log(b)*) values were analysed using generalized linear mixed-effects models (GLMM) with '*lme4*' package [7]. For the analysis, we followed the protocol of [8,9] for data exploration, model selection, and presentation. The Shapiro-Wilk test was used to test whether residuals were normally distributed. Outliers were identified using the median absolute deviation with a cut-off of 3 [10,11]. A Gamma function (experiment 1) or an Inverse Gaussian function (experiment 2) with an *identity* link transformation function were used in the GLMM. The *identity* link transformation function was used for *A* values (experiment 1) and precision values (experiment 2). An *identity* link function means that data were not transformed. However, for the *b* values (bias) we used a *log* link transformation function, thus log-transforming *b* values. For experiment 1 we chose a Gamma function for the regression analysis because most of the *A* values fell into the Gamma quantiles, allowing us to deal with the presence of outliers without removing them or transforming the original data. This is because the Gamma probability distribution allows greater variation for large mean values [8]. In general, Gamma and Inverse Gaussian distributions provided a better fit to the data because they can account for heteroscedastic patterns of increasing variability [12].

Results

Experiment 1: Control experiment for Direction Discrimination

The results of the control experiment for motion direction discrimination showed that participants, on average, needed 1.62 (SE: 0.33) training blocks to obtain the desired level of accuracy (≥ 0.95 correct performance rate). The mean accuracy at the control experiment for direction discrimination was 0.97 (SEM: 0.011). Given that the residuals were not normally distributed ($W = 0.78$, $p = 0.004$), a one-sided one-sample permutation test (sampling permutation distribution 5k) showed that accuracies on the last training block were significantly higher than a median of 0.95 ($p = 0.0378$). Additionally, we tested whether the direction of the target had any effect on performance. The residuals were not normally distributed ($W = 0.44$, $p < 0.001$). A Friedman test on performance values of the last training block did not reveal a significant effect of the target direction ($\chi^2 = 5.82$, $df = 7$, $p = 0.56$). These results suggest that after the training blocks, participants' performance remains constant over cardinal and intercardinal directions of the target.

Experiment 2: Control Experiment for Direction Discrimination and Training blocks

The results of the control experiment for motion direction discrimination showed that the mean accuracy for direction discrimination was 0.98 (SEM: 0.008), with one training block for each participant. Given that the residuals were not normally distributed ($W = 0.80$, $p = 0.027$), a one-sided one-sample permutation test (sampling permutation distribution 5k) showed that accuracies on the last training block were significantly higher than a median of 0.95 ($p = 0.0084$).

Participants, on average, performed 2.12 training blocks (SEM: 0.23). After the initial training, mean precision values across target spatial positions were: 3.65 (SEM: 0.67), 3.02 (SEM: 0.72), 3.05 (0.95), and 8.23 (SEM: 1.97) for target spatial position 1 - 4, respectively.

A series of one-sided one-sample permutation tests (sampling permutation distribution 5k) were performed for each target position on precision values to assess whether

they were significantly above zero (i.e., chance level). The results showed that all the precision values across the four serial positions were significantly above chance (all $p < 0.005$).

Experiment 2: Precision Calculation

Precision was defined as the inverse of the circular standard deviation of the angular distance (error in radians) between the target direction and the participant's response. Target and response directions (i.e., angles in radians) were wrapped to the interval [-π, π], such that odd, positive multiples of π map to π and odd, negative multiples of π map to – π. The pairwise difference around the circle between target and response angles was then computed (i.e., error in radians) and finally we calculated the inverse of the circular standard deviation of the errors.

The mean circular standard deviation was calculated on 15 measures of error for each target position and stimulation condition. However, for the No-TMS trials, the mean circular standard deviation was calculated on 30 measures of error, i.e., pooling the No-TMS trials for the hMT+ and Cz stimulation conditions. The angular distance (error) and circular standard deviation were calculated using the Matlab circular statistics toolbox (v1.21) [13,14]. Chance performance is expected to produce a precision value that approaches zero.

Modeling of Visual Short-term Memory

Delayed estimation tasks are particularly useful to assess the components of VSTM. In this account, in the variable precision (VP) model precision is variable across items and trials and previous studies have shown that visual short-term memory precision is indeed continuous and variable across memory items and trials [15-18]. In the VP model the amount of resource an item receives, thus regulating its encoding precision, varies randomly across memory items and trials and decreases with set size. The concept of resource may be linked to the gain (i.e., mean response amplitude) of a neural population pattern of activity encoding a memorized feature, such as color, orientation, motion direction etc. The higher the gain, the higher the precision with which a stimulus is encoded [17]. The variability in gain across items and trials is consistent with single neuron firing rate variability [19] and the effects of attentional fluctuations in neural populations [20,21]. Therefore, following the rationale of Fougnie et al. [16], the VP model can be considered as an infinite scale mixture, a general framework that describes error distributions with a fixed mean and a precision (scale) that is sampled from some higher-order distribution, known as the 'mixing distribution'. In fact, in the model, precision is distributed according to some higher-order distribution, for example, a truncated normal or gamma distribution. When the error is normally distributed around the correct value, and when precision (i.e., the inverse of the variance) is gamma distributed, the resulting experimental data takes the form of a generalized Student's t-distribution wrapped on the circle, when considering stimulus dimensions such as color, orientation, or motion direction. For guess rate (g), bias (μ), and SD (σ), the probability density function of this model is given by:

$$f(x;\ \mu,\sigma,g,v) = (g)\frac{1}{360} + (1-g)\psi(x;\ \mu,\sigma,v) \quad \text{Eq.5}$$

where ψ is the wrapped generalized Student's t-distribution and is:

$$\psi(x;\ \mu,\sigma,v) = \frac{c}{\sigma}\sum_{l=-\infty}^{\infty}\left(1+\frac{(x+360l-\mu)^2}{\sigma^2 v}\right)^{-(v+1)/2} \quad \text{Eq.6}$$

with

$$c = \Gamma\left(\frac{v+1}{2}\right) / \left(\Gamma\frac{v}{2}\sqrt{\pi v}\right) \quad \text{Eq.7}$$

(see [16] for more details on the VP model and mixing distribution).

The VP model we fitted to error values was characterized by three parameters: *guess rate* (*g*), the mean standard deviation of responses (*meanSD*), and the standard deviation of response error (*SDvar*). In this case, the standard deviations of observers' reports are assumed to be distributed as a normal distribution [22]. The *guess rate* (*g*) expresses the probability with which the observer does not remember the direction of the target patch

probed in the test phase and consequently guesses randomly. *MeanSD* represents the mean standard deviation of the precision of the remembered items, and it is inversely related to precision; high values in *meanSD* indicate a less precise memory representation. *SDvar* indicates intertrial variation in memory precision; high values of *SDvar* indicate high trial-to-trial variability.

The VP model was fitted to error values calculated using the circular distance (in radians) between the target RDK direction and the observer's response in the test phase. In our experiment, we tested only one set size (i.e., four memory items on each trial). The VP model was fitted using the Matlab MemToolbox [22] (http://visionlab.github.io/MemToolbox/) to assess whether there were differences in *g*, *meanSD*, and *SDvar* between the three stimulation conditions. The model also included a bias term (μ) (using the function 'WithBias' in MemToolbox), such that the central tendency of the data was not fixed at zero. However, only the parameters *g*, *meanSD*, and *SDvar* were analyzed.

The MemToolbox uses Bayesian inference to derive a probability distribution over parameter values. This probability distribution describes the reasonableness of parameters after considering the observed data considering a prior distribution (see [22] for a detailed description of model fitting and parameters estimation). The VP model was fitted to the entire set of each observer's data.

References

1. van de Ven, V.; Jacobs, C.; Sack, A.T. Topographic contribution of early visual cortex to short-term memory consolidation: A transcranial magnetic stimulation study. *J. Neurosci.* **2012**, *32*, 4–11, doi:10.1523/JNEUROSCI.3261-11.2012.
2. Pollack, I.; Norman, D.A. A non-parametric analysis of recognition experiments. *Psychon. Sci.* **1964**, *1*, 125–126.
3. Smith, W.D. Clarification of Sensitivity Measure A'. *J. Math. Psychol.* **1995**, *39*, 82–89.
4. Zhang, J.; Mueller, S.T. A note on ROC analysis and non-parametric estimate of sensitivity. *Psychometrika* **2005**, *70*, 203–212.
5. Green, D.M.S., J. A. Signal detection theory and psychophysics. Peninsula Publishing: Los Altos, CA 94023, USA, **1966**.
6. Stanislaw, H.; Todorov, N. Calculation of signal detection theory measures. *Behav. Res. Methods Instrum. Comput.* **1999**, *31*, 137–149, doi:10.3758/bf03207704.
7. Bates, D.; Mächler, M.; Bolker, B.; Walker, S. Fitting Linear Mixed-Effects Models Using lme4. *J. Stat. Softw.* **2015**, *67*, 1–48, doi:10.18637/jss.v067.i01.
8. Zuur, A.F.I., E. N.; Elphick, C. S. A protocol for data exploration to avoid common statistical problems. *Methods in Ecology and Evolution* **2010**, *1*, 3–14.
9. Zuur, A.F.I., E. N. A protocol for conducting and presenting results of regression-type analyses. *Methods in Ecology and Evolution* **2016**, *7*, 636–645.
10. Leys, C.L., C.; Klein, O.; Bernard, P.; Licata, L. Detecting outliers: Do not use standard deviation around the mean, use absolute deviation around the median. *Journal of Experimental Social Psychology* **2013**, *49*, 764–766.
11. Rousseeuw, P.J.C., C. Alternatives to the median absolute deviation. *Journal of the American Statistical Association* **1993**, *88*, 1273–1283.
12. Lo, S.; Andrews, S. To transform or not to transform: using generalized linear mixed models to analyse reaction time data. *Front Psychol* **2015**, *6*, 1171, doi:10.3389/fpsyg.2015.01171.
13. Berens, P. CircStat: A Matlab Toolbox for Circular Statistics. *Journal of Statistical Software* **2009**, *31*, doi:http://www.jstatsoft.org/v31/i10.
14. Zar, J.H. *Biostatistical Analysis*; Prentice Hall, 1996.
15. Bays, P.M.; Catalao, R.F.; Husain, M. The precision of visual working memory is set by allocation of a shared resource. *J Vis* **2009**, *9*, 7 1–11, doi:10.1167/9.10.7.
16. Fougnie, D.; Suchow, J.W.; Alvarez, G.A. Variability in the quality of visual working memory. *Nat Commun* **2012**, *3*, 1229, doi:10.1038/ncomms2237.
17. van den Berg, R.; Shin, H.; Chou, W.C.; George, R.; Ma, W.J. Variability in encoding precision accounts for visual short-term memory limitations. *Proc Natl Acad Sci U S A* **2012**, *109*, 8780-8785, doi:10.1073/pnas.1117465109.
18. van den Berg, R.; Awh, E.; Ma, W.J. Factorial comparison of working memory models. *Psychol Rev* **2014**, *121*, 124–149, doi:10.1037/a0035234.
19. Goris, R.L.; Movshon, J.A.; Simoncelli, E.P. Partitioning neuronal variability. *Nat Neurosci* **2014**, *17*, 858–865, doi:10.1038/nn.3711.
20. Cohen, M.R.; Maunsell, J.H. Attention improves performance primarily by reducing interneuronal correlations. *Nat Neurosci* **2009**, *12*, 1594–1600, doi:10.1038/nn.2439.
21. Cohen, M.R.; Maunsell, J.H. A neuronal population measure of attention predicts behavioral performance on individual trials. *J Neurosci* **2010**, *30*, 15241–15253, doi:10.1523/JNEUROSCI.2171-10.2010.

22. Suchow, J.W.; Brady, T.F.; Fougnie, D.; Alvarez, G.A. Modeling visual working memory with the MemToolbox. *J Vis* **2013**, *13*, doi:10.1167/13.10.9.
